# Dangerous neighbours: Birds and bird‐eating bats sharing tree cavities

**DOI:** 10.1002/ece3.11098

**Published:** 2024-03-11

**Authors:** Danilo Russo, Anne Mäenurm, Adriano Martinoli, Luca Cistrone

**Affiliations:** ^1^ Laboratory of Animal Ecology and Evolution (AnEcoEvo), Dipartimento di Agraria Università degli Studi di Napoli Federico II Portici Napoli Italy; ^2^ AFNI Friuli‐Venezia Giulia Cordenons Pordenone Italy; ^3^ Unità di Analisi e Gestione delle Risorse Ambientali, Guido Tosi Research Group, Dipartimento di Scienze Teoriche ed Applicate Università degli Studi dell'Insubria Varese Italy

**Keywords:** avian prey, bats, fear ecology, noctule, predation

## Abstract

Mounting evidence indicates the non‐consumptive effects of predators significantly impact prey physiology, ecology and behaviour. Passerine birds experience adverse effects on nesting and reproductive success when in proximity to predators. Fear of predators is context‐dependent and influenced by hunting habitats and foraging strategies. While some bat species prey on birds, the greater noctule (*Nyctalus lasiopterus*) stands out by specialising in avian prey, especially during peak bird migration. *N. lasiopterus* is thought to seize avian prey in flight, but direct evidence is lacking. If birds were taken from nests, they would likely avoid nesting near these bats. However, no observations support this view. This study documents the successful reproduction of Eurasian blue tits (*Cyanistes caeruleus*) nesting alongside a colony of approximately 25 greater noctules. This bird species is a prey species for greater noctules in Italy. Over about 1 month (April–May 2023), we observed parent birds provisioning food to chicks, with at least two chicks alive and fed outside the tree cavity by the end of the period. While acknowledging the limitations of a single observation, we propose that this previously unknown behaviour indirectly supports the idea that greater noctules only capture avian prey in flight, not within confined spaces. This observation challenges the perception that these bats pose a threat when sharing roosting spaces in trees, as evidenced in our observed case. We hope this novel observation inspires future research on variations in bird nesting behaviour and reproductive success in the presence of bird‐eating bats, as well as an assessment of the long‐term impact on population dynamics and reproductive success of nesting birds sharing such roosting spaces.

## Graphical abstract

Greater noctules feed on birds, including Eurasian blue tits, with the presumed strategy being in‐flight capture, yet no direct observations confirming this holds true. We recorded the successful nesting of blue tits in a tree cavity shared with around 25 greater noctules and recorded no predatory interactions over the whole nesting period. Our observation supports the hypothesis that roosting bats are not perceived as a threat, indirectly endorsing the ‘aerial hawking’ predation theory.

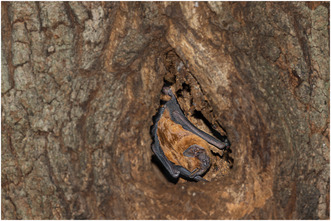

## 1 INTRODUCTION

The fear of predators is recognised as a powerful factor influencing the ecological and behavioural patterns of their prey (Orrock et al., 2013), affecting prey's vital activities such as foraging (Matassa & Trussell, 2011), resting (Stuber et al., 2014), or reproduction (LaManna & Martin, 2016). It is now well established that the interaction between predators and prey, even when non‐lethal, has significant adverse consequences on prey fitness (Clinchy et al., 2013). However, there is mounting evidence that the magnitude of non‐consumptive effects is context‐dependent, as prey may mitigate such effects by exploiting the spatial features that promote predator avoidance or sheltering (Wirsing et al., 2021). In parallel to ‘landscapes of fear’ (the spatial distribution of perceived predation risk influencing the behaviour and movements of prey species within a given area), ‘evasion landscapes’ (spatial variability in the likelihood that a predator is avoided during an encounter) are recognised. Non‐consumptive predator effects are also dependent on how predators capture their prey or their hunting strategies (Wirsing et al., 2021). It is logical to assume that if the predator–prey encounter takes place in a habitat whose structure does not allow the predator to pursue its prey, non‐consumptive effects on the latter might be much milder or nulled.

Bats exhibit a diverse range of diets globally, with 17 species incorporating vertebrates into their feeding habits (Gual‐Suárez & Medellin, 2021). Among these, nine species primarily consume terrestrial vertebrates in certain regions, with vertebrates constituting more than 50% of their prey in terms of the number, mass, volume or energy intake (Gual‐Suárez & Medellin, 2021). Notably, three species (*Nyctalus lasiopterus*, *N. aviator* and *Ia io*) seasonally target birds, particularly during bird migration or when their arthropod food sources are depleted, likely through aerial hawking (Gual‐Suárez & Medellin, 2021).

**FIGURE 1:**
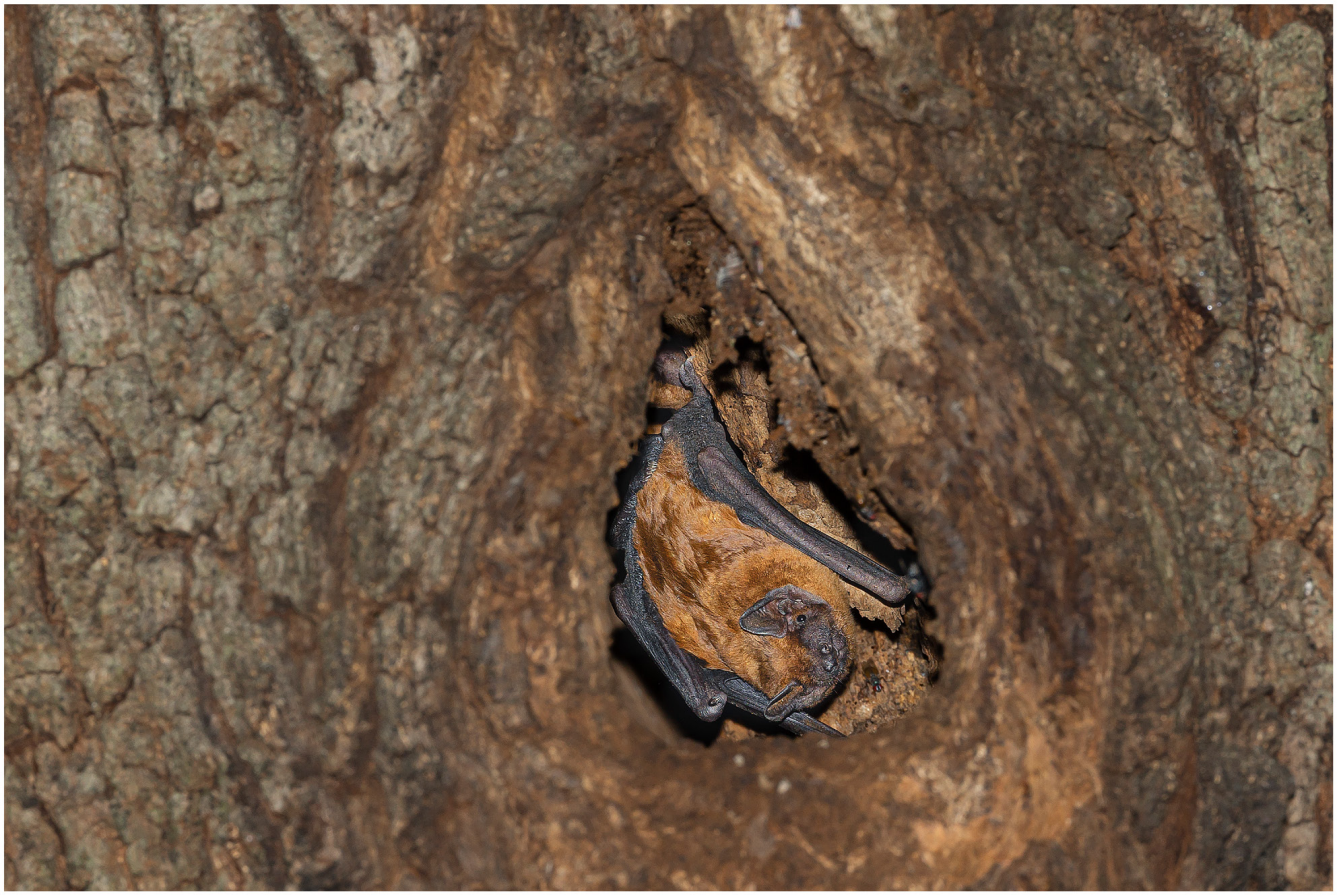
A roosting individual of greater noctule (*Nyctalus lasiopterus*). Photograph by Anne Mäenurm.

The greater noctule, *N. lasiopterus* (Figure 1) demonstrates a seasonal specialisation in birds, potentially forming the entirety of its diet during bird migration (Ibáñez et al., 2001). In summer, it predominantly feeds on insects (Smirnov & Vekhnik, 2013), although there is evidence of bird consumption even outside bird migration (Dondini & Vergari, 2000). This long‐range migratory species possesses a wing shape and echolocation call design optimised for hunting prey in flight. Unlike other carnivorous bats, such as *Vampyrum spectrum* and *Chrotopterus auritus*, which may occasionally raid bird nests (Medellín, 1988; Vehrencamp et al., 1977), *N. lasiopterus* is believed to capture birds in flight, possibly at high elevations, although direct observation is lacking (Ibáñez et al., 2001). This predation strategy would favour targeting smaller birds (less than half the predator's size) that can be easily subdued (Gual‐Suárez & Medellin, 2021). If, on the other hand, greater noctules catch birds while stationary, their preferred hunting habitat would likely be in areas where birds shelter or nest, rather than open spaces. In this scenario, narrow spaces such as roosting cavities would therefore constitute suitable hunting grounds. This ‘static’ hunting strategy would potentially result in stronger ‘non‐consumptive effects’ on the prey bird, a phenomenon highlighted by multispecies comparisons between prey reactions to stationary versus actively hunting predators (Preisser et al., 2007). Therefore, should static predation occur, birds would avoid resting or nesting near roosting greater noctules since their mere presence would elicit a strong antipredatory reaction. Conversely, should greater noctules only pursue birds on the wing, in open space, birds that are part of the bat's diet and are resting or nesting in proximity to roosting greater noctules should not interpret the predator's presence as a threat, potentially facilitating coexistence. To support this perspective, we present evidence of the successful reproduction of a pair of Eurasian blue tits (*Cyanistes caeruleus*) within the same cavity used by a greater noctule colony for roosting.

## 2 MATERIALS AND METHODS

The observation we made was part of a photographic campaign of monitoring of *N. lasiopterus* conducted between April 2021 and February 2023 in the Friuli‐Venezia Giulia Region near Udine (46.0711° N, 13.2346° E) conducted by one of us (AM). The study site is a forest island with tree cavities used as roosts by *N. lasiopterus* and *N. noctula* at an altitude of approximately 15 m a.s.l. Situated within an agricultural landscape dominated by corn, wheat and vineyards, the 20‐ha forest patch comprises diverse tree species including European aspens, European hornbeams, pedunculate oaks and others. Once part of the larger ancient forest ‘Silva lupanica’ (Mäenurm, 2021), now fragmented due to historical factors, the site provides suitable roosting cavities for bats such as noctules, which hibernate and reproduce there year‐round (Russo et al., 2023).

The surveys of roosts were conducted three to four times a week by traversing the entire forest. Roosts were identified through various methods, including the distinctive calling of *Nyctalus* colonies before sunset, daytime photographic identification of bats in cavities and observation of evening emergence from suitable roosts. The images were captured by AM using Canon EOS R and Canon EOS R6 cameras, paired with Canon 300 mm f/4 EF L USM and Canon RF 100‐500 mm f/4.5–7.1 L IS USM lenses. The photographer took precautions to stay concealed at a safe distance from the bat roosts to prevent any disturbance to their natural behaviour. No legal authorisation was required to carry out this activity. Species identification of the bats was determined through photographs taken either within the roost or during their emergence from it. Further details are provided by Russo et al. (2023).

## 3 RESULTS

**FIGURE 2:**
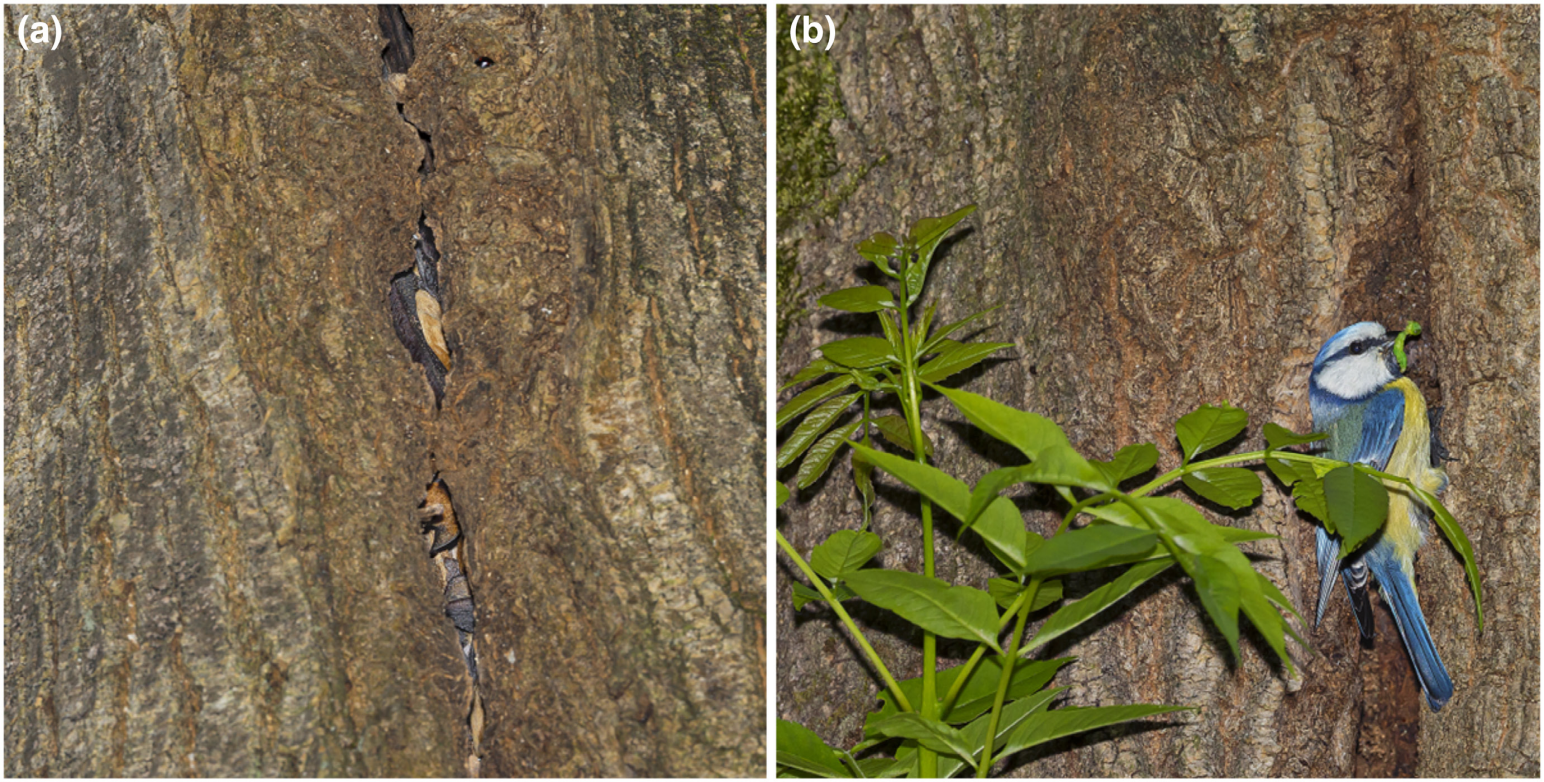
A colony of greater noctules *Nyctalus lasiopterus* (a) and nesting Eurasian blue tits *Cyanistes caeruleus* (b) using the same tree cavity in a forest island of northeast Italy, April–May 2023. Wider angle shots were intentionally omitted for conservation purposes to prevent the tree from being identifiable. Photos by Anne Mäenurm.

The roost cavity where the observation took place is a long (>1 m) vertical crack in a narrow‐leaved ash (*Fraxinus angustifolia*) which hosts bats year‐round. The cavity opens to two opposite sides of the tree and at the time the observation was made was home to a colony of approximately 25 greater noctules that roosted in the upper portion of the cavity (Figure 2). The lower section of the cavity was instead occupied by a nest of Eurasian blue tit. The base of the cavity, where the tits had nested, was about 3.8 m above the ground, while the bats roosted above the nest in the same cavity, at 15–30 cm. The cavity access was too narrow to be able to see the nest, but we observed systematically both parents bringing food to the chicks (Figure 2). For the first time, we observed parents bringing worms on April 20, 2023. Subsequently, we monitored them for a month, three times a week. The last day we observed fledglings outside the nest was on 25 May 2023, witnessing two chicks being fed by their parents. In the following days, the birds were no longer present, strongly suggesting the successful fledging of juveniles. In no case did we record interactions between the bats and the birds. The upper and lower parts of the cavity were interconnected, thereby lacking any physical barrier that would impede the bats from descending and accessing the nest. At the time when we observed bird reproduction, greater noctules were active in the roost during the daytime, as suggested by movement and chirping by individuals inside the cavity. The colony comprised males and pregnant females, with births expected in late spring or early summer.

## 4 DISCUSSION

We documented the successful reproduction of a pair of Eurasian blue tits nesting in the same cavity used by a colony of bird‐eating bats. While we refrained from using endoscopes to explore the cavity to avoid disturbance to both the bats and the nesting birds, we managed to visually inspect the cavity from a sufficiently short distance. This approach allowed us to ascertain the interconnectedness of the spaces used by the bats and the blue tits, as well as the absence of obstacles that could impede the bats from reaching the birds' nesting site.

The bird species in question is typical prey of this bat, as indicated by dietary analyses conducted in Italy (Dondini & Vergari, 2000). To the best of our knowledge, this is the first instance of potential bird prey nesting in the same roosting space as bird‐eating bats. There is solid evidence that birds are exposed to significant non‐consumptive effects of predators (Cresswell, 2008). Nesting is a highly sensitive phase of the passerine bird life cycle and is greatly affected by adverse non‐consumptive effects exerted by nearby predators (LaManna & Martin, 2016). For example, in blackbirds (*Turdus merula*), the presence of domestic cats near nests limits food provisioning to the nestlings and elicits the production of alarm calls and mobbing towards the predator, with detrimental fitness consequences. Besides implying an increase in energy expenditure, this also has the consequence of revealing the nest's presence to other opportunistic predators, increasing direct mortality (Bonnington et al., 2013).

We believe that the observation we made is a further indirect argument in support of the fact that *N. lasiopterus* captures birds on the wing rather than raiding nests, as seen in other less specialised animalivorous bats (Gual‐Suárez & Medellin, 2021). Therefore, the presence of greater noctules in the same cavity where a nest is established strongly suggests that the birds did not perceive the bat presence as a risk, since the dangerousness of the predator would rather be associated with open spaces where both prey and predator are flying.

Blue tits typically lay 7–12 eggs, with a variable fledging success rate that may reach 100% (Maícas et al., 2012). While our observation only captured two fledging birds, this may not accurately reflect overall reproductive success, given the intermittent monitoring of the nest and the possibility of additional birds fledging unnoticed. Furthermore, fledging success is influenced by various factors such as food availability (Tremblay et al., 2003), nest physical conditions (Deeming & Pike, 2015) and territorial or parental quality (Przybylo et al., 2001). To assess the real impact of greater noctule roosts on bird fitness, a controlled experiment comparing fledging success at nests near and away from bat roosts would be necessary yet extremely challenging to implement.

Interference competition for tree cavities between greater noctules and birds is documented in areas where invasive rose‐ringed parakeets (*Psittacula krameri*) occur, particularly in an urban park in southern Spain where noctules day‐roost and breed in tree cavities. In such scenarios, parakeets have been observed replacing bats in roost cavities, indicating competition through aggression. In some cases, this competition has resulted in the killing of bats, leading to adverse demographic consequences for the latter (Hernández‐Brito et al., 2014, 2018). Greater noctules are also preyed upon by avian predators, particularly tawny owls (*Strix aluco*), whose predatory pressure has been found to exert negative effects on the stability of bat colonies (Kelm et al., 2023). In all such cases, the birds involved typically have a much larger size than the bats.

We acknowledge that the case we are reporting is confined to a single nest, and it may not be entirely representative of the interactions between greater noctules and their potential avian prey at roosting sites. Nevertheless, this represents the first reported case, and we hope it will stimulate further research on the topic. Future studies might focus on variations in bird nesting behaviour and reproductive success in the presence of bird‐eating bats. Longitudinal studies are needed to assess the sustained impact of shared roosting spaces on the population dynamics and reproductive success of nesting birds. In conclusion, the intricate relationship between the bird‐eating bat, *N. lasiopterus* and its avian prey is revealed to be a multifaceted dynamic, heavily contingent on specific contexts. The observed coexistence of greater noctules and nesting birds within shared tree cavities in Italy adds another layer to our understanding. This cohabitation provides further support for the notion that *N. lasiopterus* primarily targets birds in flight and open spaces, rather than in proximity to their roosting sites and that the bat species is incapable of raiding nests. The intricate balance observed in these interactions highlights the adaptability and nuanced nature of predator–prey relationships, shedding light on the complex ecological dynamics that shape the behaviour of these enigmatic nocturnal predators.

## AUTHOR CONTRIBUTIONS

**Danilo Russo:** Conceptualization (lead); methodology (lead); supervision (lead); writing – original draft (lead). **Anne Mäenurm:** Investigation (lead); methodology (lead); writing – original draft (supporting). **Adriano Martinoli:** Funding acquisition (lead); methodology (equal); writing – original draft (equal). **Luca Cistrone:** Conceptualization (supporting); methodology (supporting); writing – original draft (supporting).

## Data Availability

All data used for this ms are presented in the text, which is based on direct observations, so they are available to editors, reviewers and all readers.
